# Forkhead box protein O1 (FoxO1) /SERPINB1 ameliorates ROS production in diabetic nephropathy

**DOI:** 10.1002/fsn3.1859

**Published:** 2020-12-20

**Authors:** Xiaoya Liang, Yanjin Su, Yongbo Huo

**Affiliations:** ^1^ Infection management department Affiliated hospital of shaanxi university of traditional Chinese medicine Xianyang Shaanxi China; ^2^ The first Department of Endocrinology Shaanxi university of traditional Chinese medicine Xianyang Shaanxi China; ^3^ Internal Medicine Department Yan'an Hospital of traditional Chinese Medicine Shaanxi Shaanxi China

**Keywords:** diabetic nephropathy, FoxO1, oxidative stress, ROS production, SERPINB1

## Abstract

With the increasing prevalence of diabetes in recent years, diabetic nephropathy (DN) has become a severe disease that greatly threatens human health. DN not only is a common complication of diabetes, but also takes an important place in kidney disease. To this end, the present study was designed to explore the effects of Forkhead box protein O1 (FoxO1) on reactive oxygen species (ROS) production in DN mice. DN mice were treated with recombinant protein of FoxO1. Afterward, inflammation ELISA kits were used to measure the levels of TNF‐α, IL‐1β, IL‐6, and IL‐18. The levels of MDA, SOD, GSH, and GSH‐PX were measured using kits according to the manufacturer's instructions. In addition, the production of ROS was assessed. Interestingly, the expression of FoxO1 was down‐regulated in DN mice. The treatment of FoxO1 recombinant protein ameliorated MDA levels, increased the levels of SOD, GSH, and GSH‐PX, and induced both mRNA and protein expression of hepatic serine protease inhibitor B1 (serpinB1) in ND mice. Similarly, FoxO1 reduced MDA levels and ROS production, increased the levels of SOD, GSH, and GSH‐PXs, and induced the mRNA and protein expression of serpinB1 in in vitro model of DN. The inhibition of serpinB1 attenuated the effects of FoxO1 on ROS production‐induced oxidative stress in in vitro model of DN. Overall, FoxO1/SERPINB1 ameliorated ROS production‐induced oxidative stress in DN.

## Graphical abstract

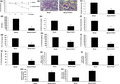

## 1 INTRODUCTION

With the increasing prevalence of diabetes in recent years, diabetic nephropathy (DN) has become a dangerous disease that threatens human health (Matsuzaki & Darcha, 2013). DN is not only a common complication of diabetes, but also takes an important place in kidney disease (Zhang, Xiong, Xiong, Liu, & Liu, 2016). The pathogenesis of DN is extremely complicated, which involves the molecular levels of human biological metabolism (Zhang et al., 2016). Therefore, it is difficult to investigate the pathogenesis of DN, without defined pathogenesis mechanism at present (Su et al., 2014). Most existing studies have shown that the pathogenesis of DN is mostly associated with heredity, abnormal glucose metabolism, abnormal lipid metabolism, microcirculation disorders, cytokine effects, inflammatory effects, and kallikrein‐kinin system (Su et al., 2014).

Oxidative stress is a primary and independent participating factor in the pathogenesis of diabetes. The major lethal complication of diabetes, namely DN, is becoming a great threat to human health (Ferreira, Robaina, Rezende, Severino, & Klumb, 2014). Recent studies have demonstrated that the roles of oxidative stress in diabetes and multiple chronic complications of diabetes (including DN) could not be ignored (Ferreira et al., 2014; Wei et al., 2015). The production of reactive oxygen species (ROS) is the basic link of oxidative stress, which can destroy tissue protein, fat, and nucleic acid reactions and simultaneously produce peroxide lipids, such as various types of aldehydes, ketones and etc., thereby amplifying oxidative injury (Zhuang et al., 2015).

FOXO1 is an important regulator of various intracellular processes, such as energy metabolism, oxidative stress response, redox signaling pathway, cell cycle progression, and apoptosis (Allison & Ditor, 2015). Other stress stimuli that can produce ROS could regulate the activation and protein expression of FOXO1 at multiple levels (Allison & Ditor, 2015). On the one hand, it can regulate the expression of FOXO1 protein at the transcription level, such as acting on the upstream regulatory protein p53 of FOXO1, affecting the regulation of miRNAs on FOXO1 mRNA, and affecting the regulatory roles of HuR on FOXO1 mRNA (Yang et al., 2014). On the other hand, it can also affect the activity, stability, and binding to DNA of FOXO1 through phosphorylation and acetylation (Freria et al., 2016; Van Straaten, Cloud, Morrow, Ludewig, & Zhao, 2014). In addition, ROS can also regulate the activation of FOXO1 protein by affecting FOXO1 transcription coactivators (Yang et al., 2014). Under some pathological conditions related to oxidative stress, the overactivation and over‐expression of FOXO1 can lead to the occurrence of various diseases, including cancer and diabetes (Yang et al., 2014).

SerpinB1 belongs to the family of serine protease inhibitors (Van Straaten et al., 2014;). The Serpin family is involved in various biochemical reactions, including regulation of coagulation (thrombosis and thrombolysis), neurotrophic factors, hormone transport, complement and inflammation, angiogenesis, and blood pressure. It has been reported that PAI‐1 (SerppinE1), Maspin (SerpinB5), SerpinB3, and SerpinB13 are all associated with angiogenesis (Freria et al., 2016; Lobenwein et al., 2015). Among them, Maspin (SerpinB5) plays a role as suppressing angiogenesis, inhibiting migration and infiltration in tumor, which is closely associated with the activation of integrin β1 (Lobenwein et al., 2015). Previous studies have found that SerpinB1 could relieve acute oxidative stress and attenuate endothelial cell apoptosis by activating the Erk1/2 pathway, thereby exerting a protective effect, demonstrating that SerpinB1 plays a key role in the proliferation of endothelial cells (Freria et al., 2016; Lobenwein et al., 2015). This study aimed to explore the effects and mechanism of FoxO1 on ROS production in diabetic nephropathy.

## 2 MATERIAL AND METHODS

### 2.1 Quantitative study of mice model

Specific pathogen‐free (SPF) grade 6‐week‐old male db/db mice were purchased from Victoria Tong Lihua (Beijing, China). This study was approved by the Institutional Animal Care and Use Committee of Affiliated hospital of shaanxi university of traditional Chinese medicine (TCM2016081601).

The animals were housed at the Laboratory Animal Center of author's affiliation at 23 ± 3°C, 55%–60% humidity under a 12 hr light/dark cycle. Mice were fed with high‐fat diet (HFD; 60% of total calories from fat, Beijing, China) for 4 months. After the mice were sacrificed, the mouse kidney tissue was removed and saved at −80°C. Number of every group was six mice. Recombinant protein of FoxO1 (1 μg/mice/week, IP) was injected model mice (Model + FoxO1 group) for 4 months, and equal volume of normal saline (IP) was injected model mice (Model group) for 4 months.

### 2.2 Microarray analysis

Microarray experiments from HK‐2 cells were performed at the Genminix Informatics (China). Gene expression was analyzed using the Human Gene Expression 4x44K v2 Microarray Kit (Agilent, Santa Clara, CA). Data were obtained using the Agilent Feature Extraction software.

### 2.3 Cell culture and transfection

HK‐2 cells were purchased from Shanghai cell bank, Chinese academy of sciences and were grown in F12/ DMEM supplemented with 10% (*v*/v) fetal calf serum (FCS, Gibco, NY, USA) at 37°C in 95% humidified air and 5% CO_2_. Cell was transfected using Lipofectamine 2000 (Invitrogen, USA) with FoxO1, siFoxO1, SERPINB1 siSERPINB1, and negative mimics for 24 hr. After transfection, HK‐2 cells were incubated with high glucose (40.9 mM) for 48 hr.

### 2.4 Measurement of inflammation, intracellular oxidative stress, and ROS detection

TNF‐α (H052), IL‐1β (H002), IL‐6 (H007), and IL‐18 (H015) levels from mice kidney tissue and HK‐2 cells were measured using inflammation ELISA Kits (Nanjing Jiancheng Bioengineering Research Institute Co., Ltd, Nanjing, China). MDA (A003‐1‐2), SOD (A001‐3‐2), GSH (A006‐2‐1), and GSH‐PX (A005‐1‐2) levels from mice kidney tissue and HK‐2 cells were measured using MDA, SOD, GSH, and GSH‐PX kits according to the methods of manufacturer instructions (Nanjing Jiancheng Bioengineering Research Institute Co., Ltd, Nanjing, China). Absorbance was measured using UniCel DxC 600 Chemistry Analyzer (Beckman Coulter, CA, USA). ROS production from HK‐2 cells was assessed using an Olympus FluoView1000 confocal laser scanning microscope (using Ex/Em *λ* = 480 nm/535 nm).

### 2.5 Western blotting

Total protein was extracted from mice kidney tissue and HK‐2 cells with a RIPA lysis solution, and protein content was measured using BCA assay. An equal amount of protein was separated using SDSPAGE and then transferred to a nitrocellulose membrane. Membrane was blocked using 5% skim milk for 2 hr at room temperature and incubated with primary antibodies: FoxO1 (ab179450, 1:1000, abcam), SERPINB1 (ab181084, 1:1000, abcam) and GAPDH (sc‐47724, 1:5000, Santa Cruz Biotechnology) at 4°C over‐night. Membrane was incubated with peroxidase‐labeled secondary antibodies at 37°C for 1 hr. The protein bands were observed using ECL assay and quantified with the ChemiDoc XRS + imaging system (Bio‐Rad, Hercules, CA, USA).

### 2.6 Histology studies

Kidney tissues samples were fixed with 4% paraformaldehyde for 24 hr. 4‐µm‐thick sections of kidney tissues were embedded in paraffin and stained with hematoxylin–eosin(H‐E) and periodic acid–Schiff (PAS) staining. Kidney tissue samples were observed and imaged under a Leica microscope. Tubular injury score was calculated as follows: 0 = none, 1  =  ≤10%; 2 = 11%–25%; 3 = 26%–45%; 4 = 46%–75%; and 5  =  >76%.

### 2.7 Statistical analysis

The statistical software SPSS 21.0 was used for the analysis. The data are represented as the mean ± standard error of the mean (*SEM*). One‐way ANOVA or Student's *t* test was performed for data from multiple groups or two groups. Differences were considered statistically significant at *p* < .05.

## 3 RESULTS

### 3.1 The protective effect of FoxO1 in DN

**Figure 1:**
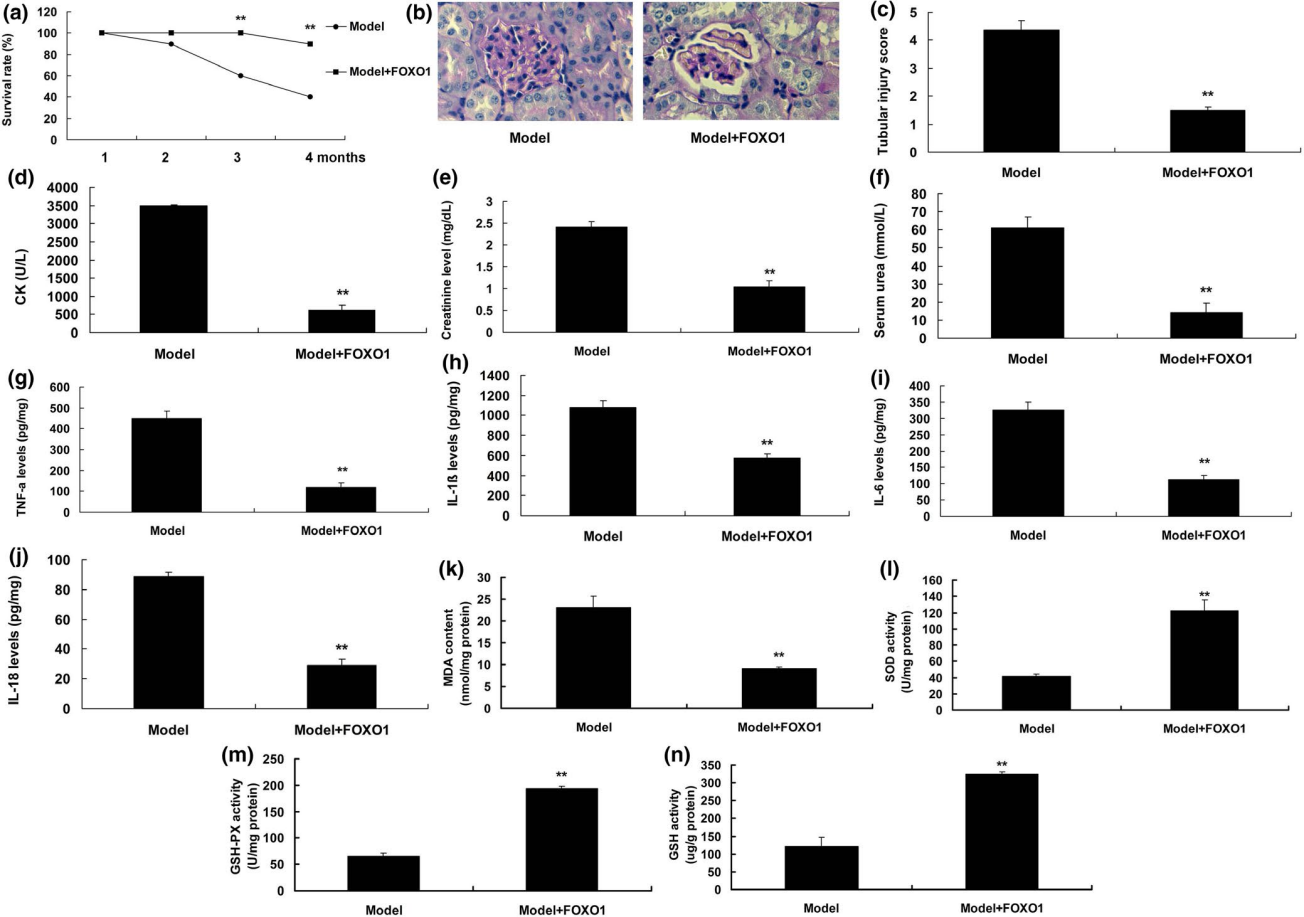
The protective effect of FoxO1 in diabetic nephropathy. Survival rate (a), kidney beans by HE (b), tubular injury score (c), CK levels (d), Creatinine (e), serum urea (f), TNF‐α, IL‐1β, IL‐6, and IL‐18 levels (g, h, i, and j), MDA, SOD, GSH, and GSH‐PX levels (K, L, M, and N). Model, diabetic nephropathy model group; Model + FoxO1, diabetic nephropathy model by FoxO1 (1 ng/kg) group. **p < .01 compared with diabetic nephropathy model group

To systematically identify the protective effects of FoxO1 in DN, DN mice were treated with recombinant protein of FoxO1. The survival rate in DN model group was higher than that of FoxO1 treatment group (Figure 1a). HE and PAS staining showed the form of kidney beans recovered and renal fibrosis was reduced in FoxO1 treatment group, comparing to model mice group (Figure 1b). Creatinine, serum urea and CK levels, and tubular injury score in model of acute renal injury were higher than those of sham group (Figure 1c‐f). Meanwhile, the levels of TNF‐α, IL‐1β, IL‐6, and IL‐18 in FoxO1 treatment mice were decreased, compared with DN model group (Figure 1g‐j). However, FoxO1 reduced MDA levels and increased the levels of SOD, GSH, and GSH‐PX in DN model mice (Figure 1k‐n).

### 3.2 FoxO1 reduced ros production in vitro

**Figure 2:**
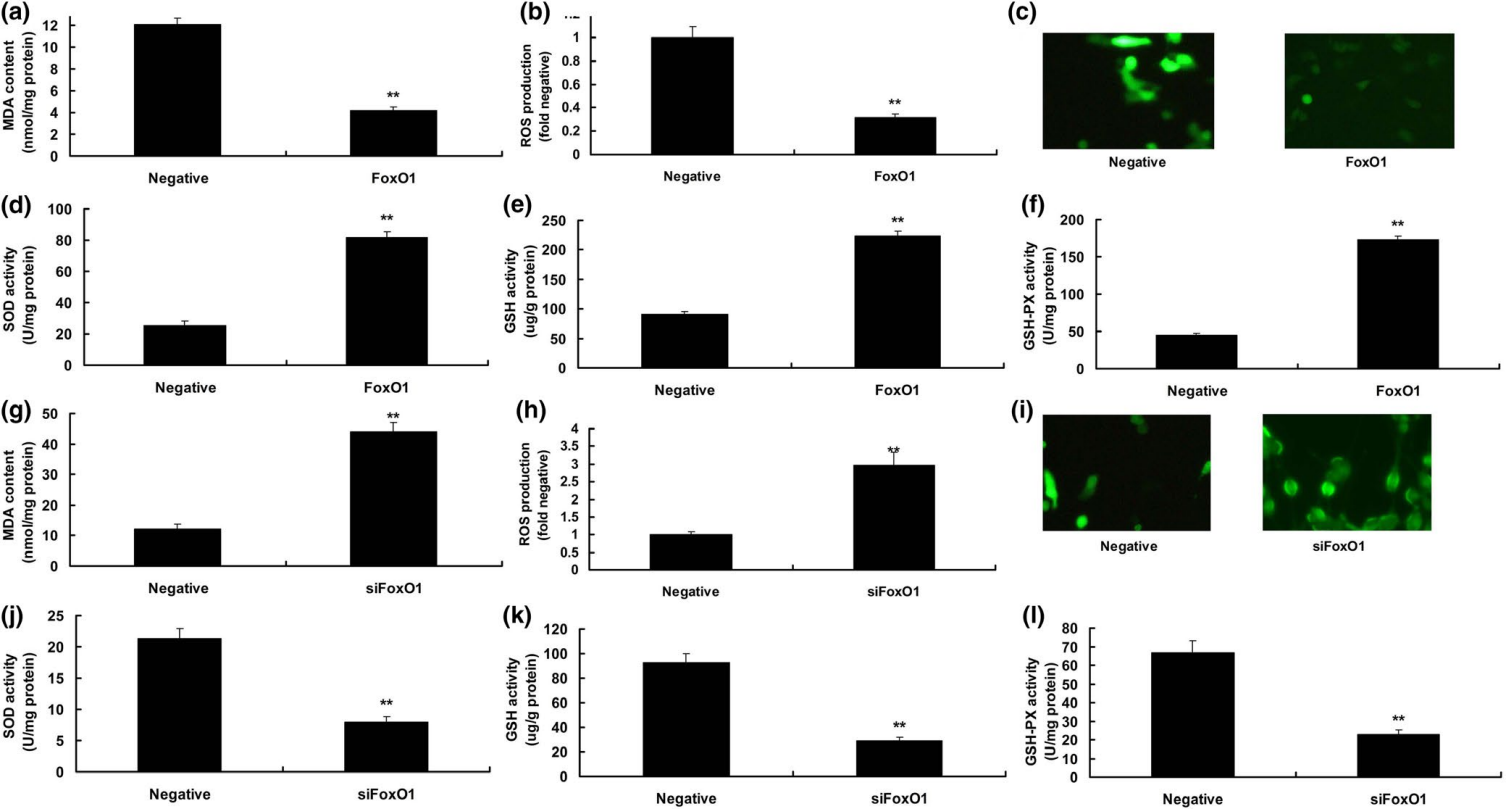
FoxO1 reduced ROS production in vitro model. MDA (a), ROS production (b and c), SOD (d), GSH (e) and GSH‐PX (f) levels by over‐expression of FoxO1; MDA (g), ROS production (h and i), SOD (j), GSH (k) and GSH‐PX (l) levels by down‐regulation of FoxO1. Negative, negative mimics group; FoxO1, up‐regulation of FoxO1 expression group; siFoxO1, down‐regulation of FoxO1 expression group. **p < .01 compared with negative mimics group

To determine the mechanism of FoxO1 on ROS production in in vitro model of DN, over‐expression of FoxO1 reduced MDA levels and ROS production levels, and enhanced the levels of SOD, GSH, and GSH‐PX in in vitro model of DN, compared to the in vitro model of DN group (Figure 2a‐f). In addition, down‐regulation of FoxO1 enhanced MDA levels and ROS production, and suppressed the levels of SOD, GSH, and GSH‐PX in in vitro model of DN, in comparison with the in vitro model of DN group (Figure 2g‐l).

### 3.3 FoxO1 regulated serpinb1 to reduce ros production in DN

**Figure 3:**
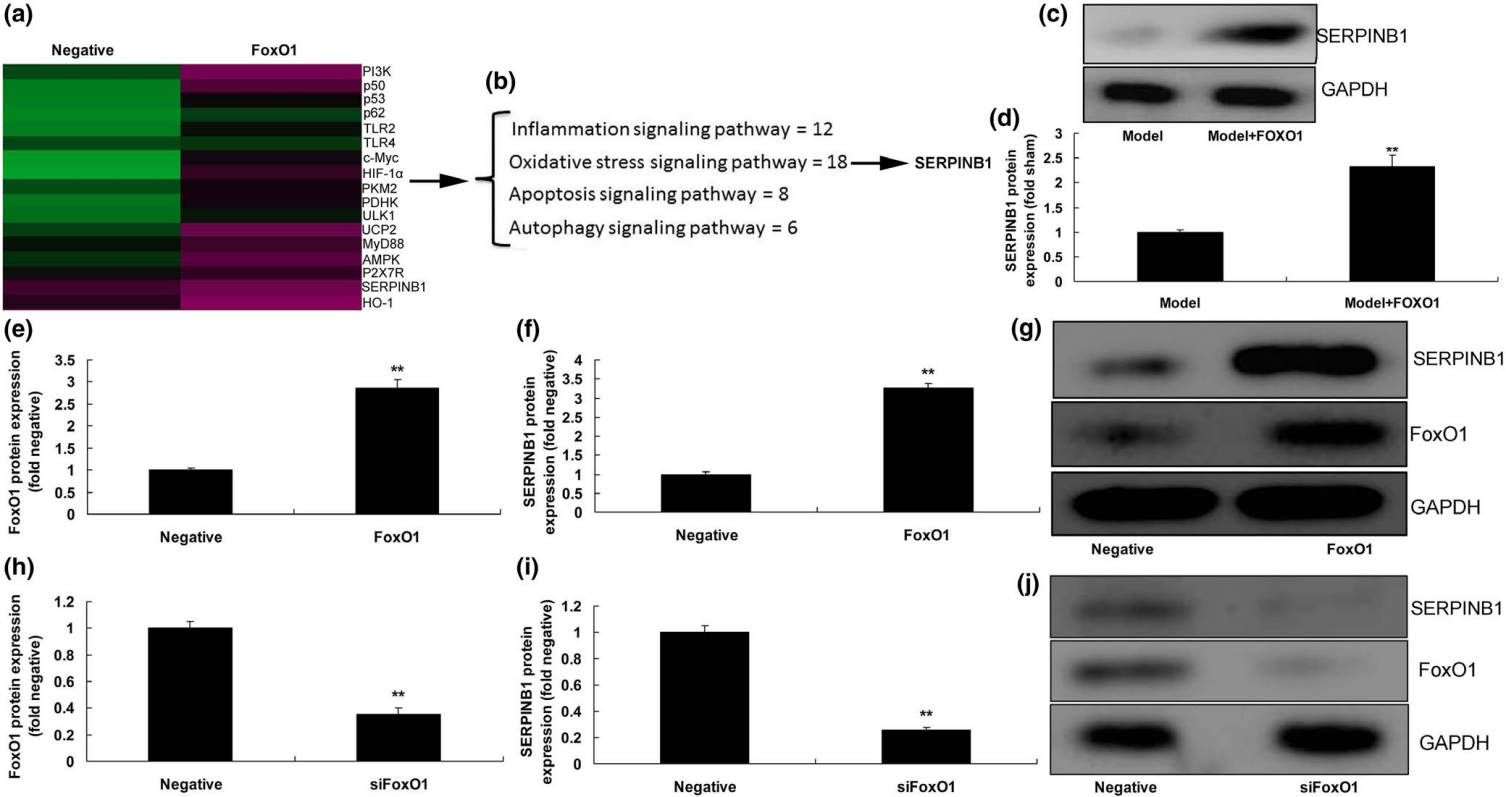
FoxO1 regulated SERPINB1 to reduce ROS production in diabetic nephropathy. Heat map (a), analysis results (b), SERPINB1 protein expression (c and d) in mice of diabetic nephropathy; FoxO1 and ERPINB1 protein expression (e, f, and g) in vitro model of diabetic nephropathy by over‐expression of FoxO1; FoxO1, and ERPINB1 protein expression (h, i, and j) in vitro model of diabetic nephropathy by down‐regulation of FoxO1. Negative, negative mimics group; FoxO1, up‐regulation of FoxO1 expression group; siFoxO1, down‐regulation of FoxO1 expression group. **p < .01 compared with negative mimics group

To determine the mechanism of FoxO1 on ROS production in in vitro model of DN, gene chip showed that FoxO1 may regulate SERPINB1 expression (Figure 3a). In DN mice, FoxO1 induced the protein expression of SERPINB1, compared to model mice of DN group (Figure 3c‐d). In vitro model of DN, over‐expression of FoxO1 induced the protein expression of FoxO1 and SERPINB1 (Figure 3e‐g). However, down‐regulation of FoxO1 suppressed the protein expression of FoxO1 and SERPINB1 (Figure 3h‐j).

### 3.4 Serpinb1 reduced ros production in vitro

**Figure 4:**
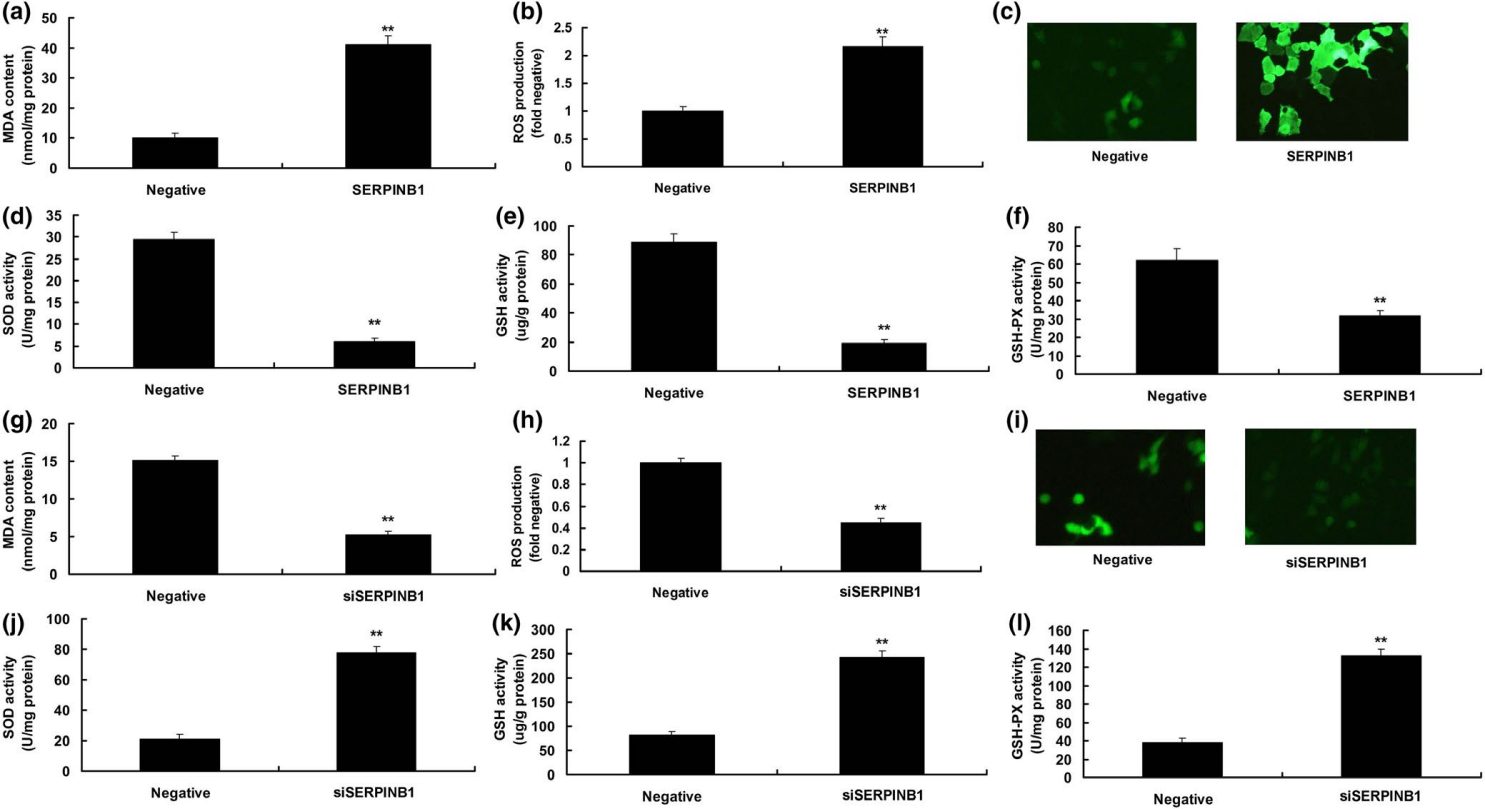
SERPINB1 reduced ROS production in vitro model. MDA (a), ROS production (b and c), SOD (d), GSH (e), and GSH‐PX (f) levels by over‐expression of SERPINB1; MDA (g), ROS production (h and i), SOD (j), GSH (k) and GSH‐PX (l) levels by down‐regulation of SERPINB1. Negative, negative mimics group; SERPINB1, up‐regulation of SERPINB1 expression group; siSERPINB1, down‐regulation of SERPINB1 expression group. **p < .01 compared with negative mimics group

To determine the effects of SERPINB1 on ROS production in in vitro model of DN, over‐expression of SERPINB1 reduced MDA levels and ROS production levels and enhanced the levels of SOD, GSH, and GSH‐PX in in vitro model of diabetic nephropathy, compared to the in vitro model of DN group (Figure 4a‐f). Down‐regulation of SERPINB1 enhanced MDA levels and ROS production, and suppressed the levels of SOD, GSH, and GSH‐PX in in vitro model of DN, in comparison with the in vitro model of DN group (Figure 4g‐l).

### 3.5 The inhibition of SERPINB1 attenuated the effects of FoxO1 in vitro

**Figure 5:**
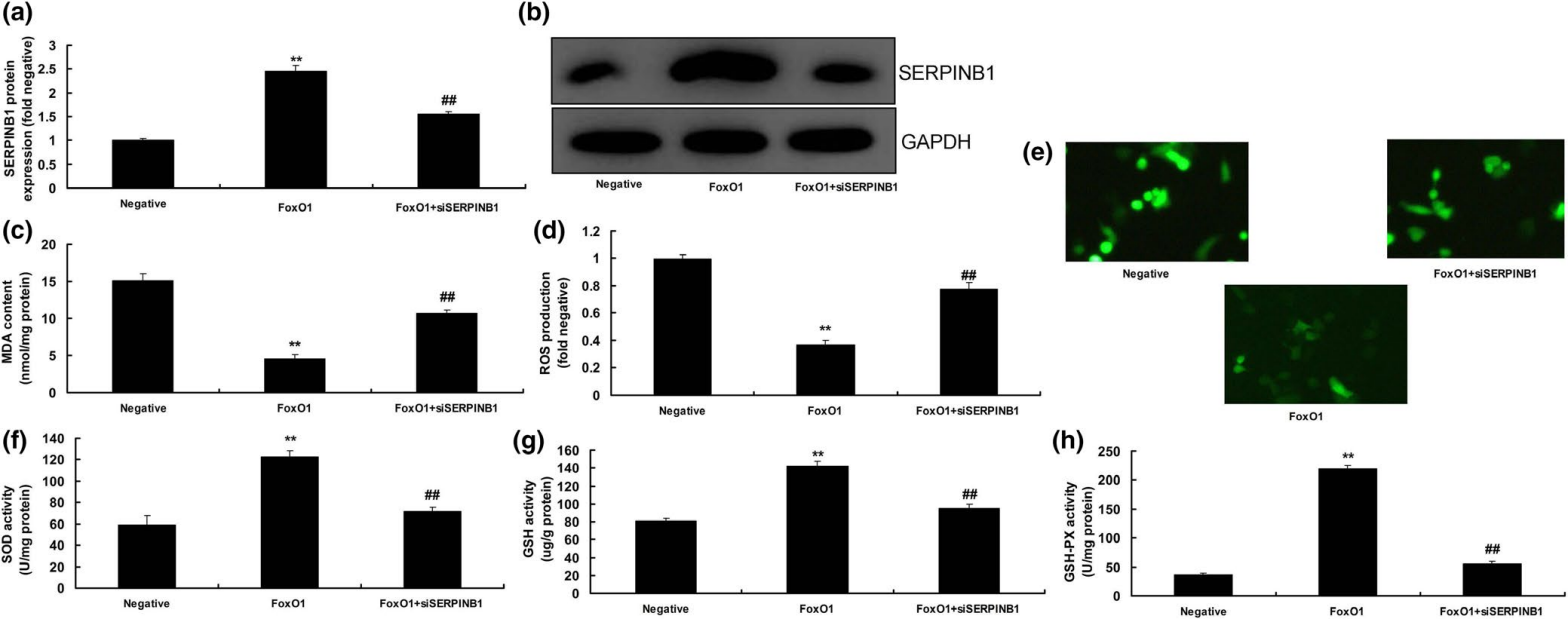
The inhibition of SERPINB1 reduced the effects of FoxO1 in vitro model. SERPINB1 protein expression (a and b), MDA (c), ROS production (d and e), SOD (f), GSH (g), and GSH‐PX (h) levels. Negative, negative mimics group; FoxO1, up‐regulation of FoxO1 expression group; FoxO1 + siSERPINB1, up‐regulation of FoxO1 expression and down‐regulation of SERPINB1 group; **p < .01 compared with negative mimics group, ##*p* < .01 compared with up‐regulation of FoxO1 expression group

The study further determined the function of SERPINB1 in the effects of FoxO1 in vitro. siSERPINB1 was used to suppress the protein expression of SERPINB1 in in vitro model of DN by over‐expression of FoxO1 (Figure 5a‐b). In addition, siSERPINB1 reduced MDA levels and ROS production, and enhanced the levels of SOD, GSH, and GSH‐PX in in vitro model of DN by over‐expression of FoxO1 (Figure 5c‐h).

### 3.6 The activation of SERPINB1 attenuated the effects of siFoxO1 in vitro

**Figure 6:**
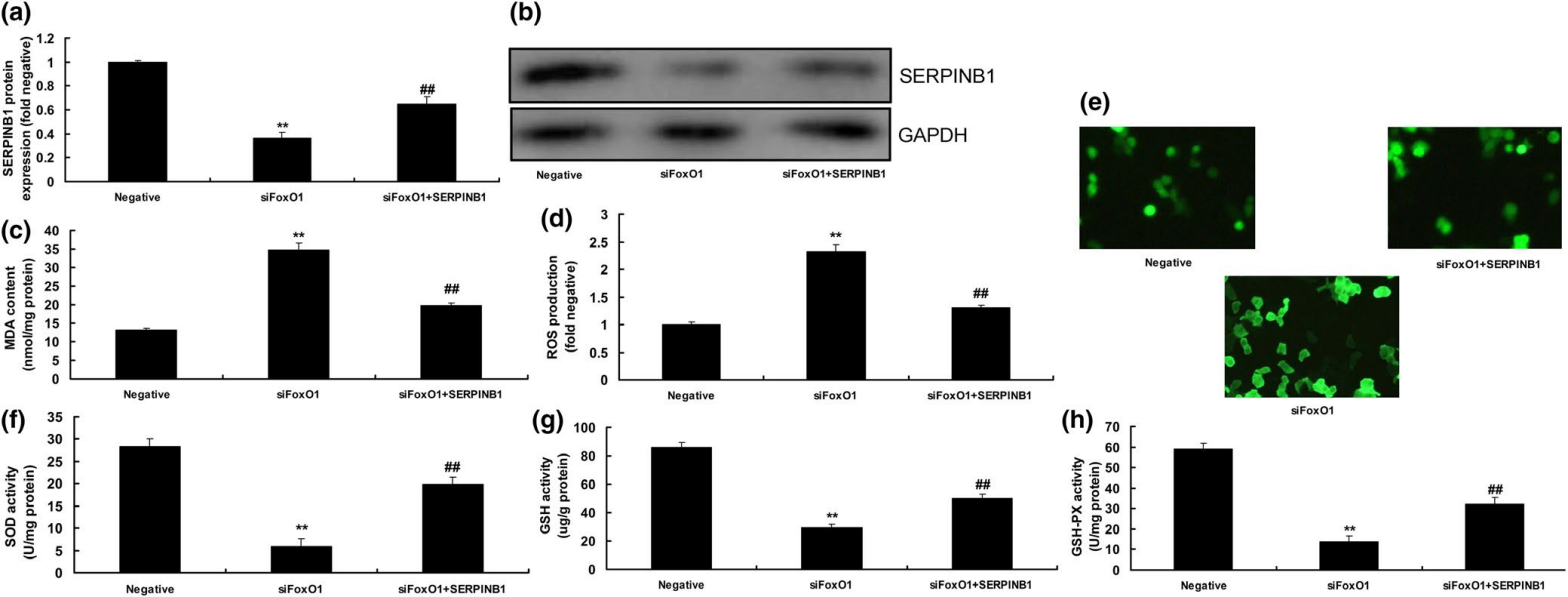
The activation of SERPINB1 reduced the effects of siFoxO1 in vitro model. SERPINB1 protein expression (a and b), MDA (c), ROS production (d and e), SOD (f), GSH (g), and GSH‐PX (h) levels. Negative, negative mimics group; siFoxO1, down‐regulation of FoxO1 expression group; siFoxO1 + SERPINB1, down‐regulation of FoxO1 expression and up‐regulation of SERPINB1 group; **p < .01 compared with negative mimics group, ##*p* < .01 compared with down‐regulation of FoxO1 expression group

SERPINB1 plasmid was used to induce SERPINB1 protein expression in in vitro model of DN by down‐regulation of FoxO1 (Figure 6a‐b). As a result, over‐expression of SERPINB1 enhanced MDA levels and ROS production, and suppressed the levels of **SOD, GSH, and GSH‐PX** in in vitro model of DN by down‐regulation of FoxO1 (Figure 6c‐6h).

## 4 DISCUSSION

DN is one of the major chronic complications of Type 1 and Type 2 diabetes, which is also the leading cause of death in patients with Type 1 diabetes (Zhao, Liu, Dong, & Xiong, 2017). The pathogenesis of DN is extremely complex, which is currently considered as a comprehensive consequence of multiple factors based on genetic background (Xia et al., 2014). Accumulative in vivo and in vitro experiments have confirmed that oxidative stress is significantly increased in diabetes (Zhao et al., 2017). ROS produced in oxidative stress plays a key role in the occurrence and development of DN, which might be one of its main pathogenesis. The investigations into oxidative stress are extremely active (Nussbaum et al., 2013). This study found that FoxO1 reduced oxidative stress in diabetic nephropathy vivo or vitro model. Michael et al. showed that serpinB1 is related to insulin sensitivity in non‐diabetic adults (Deng et al., 2015). Based on these data, serpinB1 may protect renal cells against ROS production in diabetic nephropathy.

The activity of FOXO is controlled by various post‐transcriptional modifications and nucleoplasmic shuttles (Celik, Erhan, Gunduz, & Lakse, 2013). Recently, the regulation of post‐transcriptional modification of FOXO synthesis has become a new regulatory pattern of FOXO function (Celik et al., 2013). The post‐transcriptional modification regulatory patterns of FOXO are mainly used in response to stress stimuli, including oxidative stress (Apostolidis, Thompson, Yan, & Mourad, 2013). FOXO is a substrate for various kinases and can be phosphorylated, acetylated, ubiquitinated, glycosylated, and oxidized. These modifications can affect the activity of FOXO, such as DNA binding, transcriptional activation activity, subunit localization, binding to transcription co‐regulators of FOXO (Yang et al., 2012). This study reported that FoxO1 regulated SERPINB1 to reduce ROS production in diabetic nephropathy. Abdelfattah et al. indicated that FoxO1 promotes serpinB1 expression in hepatic insulin resistance (Liu et al., 2015).

Serpin B1 is a member of the family of serine protease inhibitor family and mainly protects tissue cells from protease damage in the cytoplasm during stress (Liang et al., 2013). Serpin B1 has been reported to be involved in inflammation (Li et al., 2014). The massive expression of Serpin B1 in inflammatory cells is involved in the pathogenesis mechanism of pulmonary inflammation. Studies on patients with ulcerative colitis have demonstrated the expression of SerpinB1 not only in inflammatory infiltrating cells, but also in colonic epithelial cells (Li et al., 2014; Liang et al., 2015). In addition, this study showed that the inhibition of SERPINB1 reduced the effects of FoxO1 in vitro model. Yao et al. reported that SERPINB1 ameliorates acute lung injury via dependent HO‐1‐oxidative stress (Wadsworth, Haines, Cornwell, Rodwell, & Paratz, 2012), demonstrating FoxO1/SERPINB1 ameliorates ROS production in diabetic nephropathy.

## 5 STUDY LIMITATIONS

The detailed mechanism by which FoxO1/SERPINB1 ameliorates ROS production in diabetic nephropathy needs further investigation.

## 6 CONCLUSIONS

Taken together, the results showed that FoxO1/SERPINB1 ameliorates ROS production—induced oxidative stress in diabetic nephropathy vivo or vitro model. The present study provides some ideas for FoxO1/SERPINB1 that can relieve the symptoms of ROS production in diabetic nephropathy.

## CONFLICTS OF INTEREST

None.

## **AUTHOR** **CONTRIBUTIONS**

Xiaoya Liang, Yanjin Su, Yongbo Huo served as a guarantor of integrity of the entire study, involved in study concepts, study design, literature research, clinical studies, experimental studies, manuscript editing, manuscript preparation, statistical analysis, data analysis, and data acquisition, and defined the intellectual content. Xiaoya Liang, Yongbo Huo involved in manuscript review.

## ETHICAL APPROVAL

This study was approved by the Institutional Animal Care and Use Committee of Affiliated hospital of shaanxi university of traditional Chinese medicine.

## Data Availability

The datasets used or analyzed during the current study are available from the corresponding author on reasonable request.

## References

[fsn31859-bib-0001] Allison, D. J. , & Ditor, D. S. (2015). Targeting inflammation to influence mood following spinal cord injury: A randomized clinical trial. Journal of Neuroinflammation, 12, 204 10.1186/s12974-015-0425-2 26545369 PMC4636770

[fsn31859-bib-0002] Apostolidis, A. , Thompson, C. , Yan, X. , & Mourad, S. (2013). An exploratory, placebo‐controlled, dose‐response study of the efficacy and safety of onabotulinumtoxinA in spinal cord injury patients with urinary incontinence due to neurogenic detrusor overactivity. World Journal of Urology, 31(6), 1469–1474. 10.1007/s00345-012-0984-0 23160758

[fsn31859-bib-0003] Celik, E. C. , Erhan, B. , Gunduz, B. , & Lakse, E. (2013). The effect of low‐frequency TENS in the treatment of neuropathic pain in patients with spinal cord injury. Spinal Cord, 51(4), 334–337. 10.1038/sc.2012.159 23295472

[fsn31859-bib-0004] Deng, X. , Zhang, Y. , Jiang, F. , Chen, R. , Peng, P. , Wen, B. , & Liang, J. (2015). The Chinese herb‐derived Sparstolonin B suppresses HIV‐1 transcription. Virology Journal, 12, 108 10.1186/s12985-015-0339-8 26206295 PMC4513614

[fsn31859-bib-0005] Ferreira, A. C. , Robaina, M. C. , Rezende, L. M. , Severino, P. , & Klumb, C. E. (2014). Histone deacetylase inhibitor prevents cell growth in Burkitt's lymphoma by regulating PI3K/Akt pathways and leads to upregulation of miR‐143, miR‐145, and miR‐101. Annals of Hematology, 93(6), 983–993. 10.1007/s00277-014-2021-4 24577510

[fsn31859-bib-0006] Freria, C. M. , Bernardes, D. , Almeida, G. L. , Simões, G. F. , Barbosa, G. O. , & Oliveira, A. L. (2016). Impairment of toll‐like receptors 2 and 4 leads to compensatory mechanisms after sciatic nerve axotomy. Journal of Neuroinflammation, 13(1), 118 10.1186/s12974-016-0579-6 27222120 PMC4879730

[fsn31859-bib-0007] Li, X. Q. , Lv, H. W. , Tan, W. F. , Fang, B. , Wang, H. , & Ma, H. (2014). Role of the TLR4 pathway in blood‐spinal cord barrier dysfunction during the bimodal stage after ischemia/reperfusion injury in rats. Journal of Neuroinflammation, 11, 62 10.1186/1742-2094-11-62 24678770 PMC3977699

[fsn31859-bib-0008] Liang, Q. , Dong, S. , Lei, L. , Liu, J. , Zhang, J. , Li, J. , … Fan, D. (2015). Protective effects of Sparstolonin B, a selective TLR2 and TLR4 antagonist, on mouse endotoxin shock. Cytokine, 75(2), 302–309. 10.1016/j.cyto.2014.12.003 25573805 PMC4950682

[fsn31859-bib-0009] Liang, Q. , Yu, F. , Cui, X. , Duan, J. , Wu, Q. , Nagarkatti, P. , & Fan, D. (2013). Sparstolonin B suppresses lipopolysaccharide‐induced inflammation in human umbilical vein endothelial cells. Archives of Pharmacal Research, 36(7), 890–896. 10.1007/s12272-013-0120-8 23604718 PMC4145723

[fsn31859-bib-0010] Liu, Q. , Li, J. , Liang, Q. , Wang, D. , Luo, Y. , Yu, F. , … Fan, D. (2015). Sparstolonin B suppresses rat vascular smooth muscle cell proliferation, migration, inflammatory response and lipid accumulation. Vascular Pharmacology, 67–69, 59–66. 10.1016/j.vph.2015.03.015 PMC4433853 25869499

[fsn31859-bib-0011] Lobenwein, D. , Tepeköylü, C. , Kozaryn, R. , Pechriggl, E. J. , Bitsche, M. , Graber, M. , … Holfeld, J. (2015). Shock wave treatment protects from neuronal degeneration via a toll‐like receptor 3 dependent mechanism: implications of a first‐ever causal treatment for ischemic spinal cord injury. Journal of the American Heart Association, 4(10), e002440 10.1161/JAHA.115.002440 26508745 PMC4845137

[fsn31859-bib-0012] Matsuzaki, S. , & Darcha, C. (2013). In vitro effects of a small‐molecule antagonist of the Tcf/ß‐catenin complex on endometrial and endometriotic cells of patients with endometriosis. PLoS One, 8(4), e61690 10.1371/journal.pone.0061690 23626717 PMC3634014

[fsn31859-bib-0013] Nussbaum, E. L. , Flett, H. , Hitzig, S. L. , McGillivray, C. , Leber, D. , Morris, H. , & Jing, F. (2013). Ultraviolet‐C irradiation in the management of pressure ulcers in people with spinal cord injury: A randomized, placebo‐controlled trial. Archives of Physical Medicine and Rehabilitation, 94(4), 650–659. 10.1016/j.apmr.2012.12.003 23246896

[fsn31859-bib-0014] Su, J. , Liang, H. , Yao, W. , Wang, N. , Zhang, S. , Yan, X. , … Wang, Y. (2014). MiR‐143 and MiR‐145 regulate IGF1R to suppress cell proliferation in colorectal cancer. PLoS One, 9(12), e114420 10.1371/journal.pone.0114420 25474488 PMC4256231

[fsn31859-bib-0015] Van Straaten, M. G. , Cloud, B. A. , Morrow, M. M. , Ludewig, P. M. , & Zhao, K. D. (2014). Effectiveness of home exercise on pain, function, and strength of manual wheelchair users with spinal cord injury: A high‐dose shoulder program with telerehabilitation. Archives of Physical Medicine and Rehabilitation, 95(10), 1810–1817.e2. 10.1016/j.apmr.2014.05.004 24887534 PMC4182115

[fsn31859-bib-0016] Wadsworth, B. M. , Haines, T. P. , Cornwell, P. L. , Rodwell, L. T. , & Paratz, J. D. (2012). Abdominal binder improves lung volumes and voice in people with tetraplegic spinal cord injury. Archives of Physical Medicine and Rehabilitation, 93(12), 2189–2197. 10.1016/j.apmr.2012.06.010 22732370

[fsn31859-bib-0017] Wei, J. , Ma, Z. , Li, Y. , Zhao, B. , Wang, D. , Jin, Y. , & Jin, Y. (2015). miR‐143 inhibits cell proliferation by targeting autophagy‐related 2B in non‐small cell lung cancer H1299 cells. Molecular Medicine Reports, 11(1), 571–576. 10.3892/mmr.2014.2675 25322940

[fsn31859-bib-0018] Xia, H. , Sun, S. , Wang, B. , Wang, T. , Liang, C. , Li, G. , … Chu, X. (2014). miR‐143 inhibits NSCLC cell growth and metastasis by targeting Limk1. International Journal of Molecular Sciences, 15(7), 11973–11983. 10.3390/ijms150711973 25003638 PMC4139824

[fsn31859-bib-0019] Yang, M. L. , Li, J. J. , Gao, F. , Du, L. J. , Zhao, H. P. , Wang, Y. M. , … Zhou, T. J. (2014). A preliminary evaluation of the surgery to reconstruct thoracic breathing in patients with high cervical spinal cord injury. Spinal Cord, 52(7), 564–569. 10.1038/sc.2014.64 24861703

[fsn31859-bib-0020] Yang, M. L. , Li, J. J. , So, K. F. , Chen, J. Y. , Cheng, W. S. , Wu, J. , … Young, W. (2012). Efficacy and safety of lithium carbonate treatment of chronic spinal cord injuries: A double‐blind, randomized, placebo‐controlled clinical trial. Spinal Cord, 50(2), 141–146. 10.1038/sc.2011.126 22105463

[fsn31859-bib-0021] Zhang, L. , Xiong, W. , Xiong, Y. , Liu, H. , & Liu, Y. (2016). 17 β‐Estradiol promotes vascular endothelial growth factor expression via the Wnt/β‐catenin pathway during the pathogenesis of endometriosis. Molecular Human Reproduction, 22(7), 526–535. 10.1093/molehr/gaw025 27009232

[fsn31859-bib-0022] Zhao, L. , Liu, L. , Dong, Z. , & Xiong, J. (2017). miR‐149 suppresses human non‐small cell lung cancer growth and metastasis by inhibiting the FOXM1/cyclin D1/MMP2 axis. Oncology Reports, 38(6), 3522–3530. 10.3892/or.2017.6047 29130108

[fsn31859-bib-0023] Zhuang, M. , Shi, Q. , Zhang, X. , Ding, Y. , Shan, L. , Shan, X. , … Shu, Y. (2015). Involvement of miR‐143 in cisplatin resistance of gastric cancer cells via targeting IGF1R and BCL2. Tumour Biology: The Journal of the International Society for Oncodevelopmental Biology and Medicine, 36(4), 2737–2745. 10.1007/s13277-014-2898-5 25492481

